# An Exceptional Primary Breast Tumor: A Report of Two Mucinous Cystadenocarcinoma Cases

**DOI:** 10.7759/cureus.95280

**Published:** 2025-10-24

**Authors:** Saad Assila, Youssef Mahdi, Leila Benbella, Mouna Khmou, Basma Elkhannoussi

**Affiliations:** 1 Department of Pathology, Mohammed V Military Instruction Hospital, Rabat, MAR; 2 Department of Pathology, Faculty of Medicine and Pharmacy, Mohammed V University, Rabat, MAR; 3 Department of Pathology, National Institute of Oncology, Ibn Sina University Hospital Center, Rabat, MAR

**Keywords:** breast, mucinous cystadenocarcinoma, mucin-producing tumors, rare cancer, triple negative breast cancer (tnbc)

## Abstract

Mucinous cystadenocarcinoma of the breast (BMCA) is an exceptionally rare malignant tumor. Histologically, BMCA features cystic structures lined with tall columnar cells rich in intracytoplasmic and extracellular mucin, resembling mucinous cystadenocarcinomas of the ovary, pancreas, and appendix. Typically triple-negative for hormone receptors and human epidermal growth factor receptor 2 (HER2), BMCA paradoxically demonstrates a better prognosis compared to other triple-negative breast cancers. Diagnosis requires exclusion of metastatic mucinous carcinomas from other primary sites and differentiation from other mucin-producing breast tumors through a combination of clinical, radiological, histopathological, and immunohistochemical evaluations. We present two cases illustrating the variable histological features and hormone receptor status of BMCA, emphasizing differential diagnosis challenges. Given its rarity, further studies are necessary to better clarify the molecular pathogenesis of BMCA, guiding optimal management strategies.

## Introduction

With fewer than 50 case reports documented in the English literature by May 2025 [1], mucinous cystadenocarcinoma of the breast (BMCA) is considered a rare malignant tumor. First identified by Koenig and Tavasoli in 1998 [2], it is acknowledged as a distinct entity of breast cancer in the 2019 World Health Organization classification (WHO) of breast tumors [3]. BMCA exhibits distinctive histopathological features marked by cystic structures lined by tall columnar cells that contain abundant intracytoplasmic and extracytoplasmic mucin, resembling its more common counterparts found in the ovary, pancreas, and appendix [2,4,5]. BMCA is usually negative for estrogen receptor (ER), progestogen receptor (PR), and human epidermal growth factor receptor 2 (HER2) expression [5,6]. Nevertheless, its prognosis is more favorable compared to other triple-negative breast cancers (TNBCs) [6,7]. When a breast tumor is histologically consistent with mucinous cystadenocarcinoma, it is essential to exclude metastasis and other mucus-secreting breast cancers [5,6]. Consequently, the differential diagnosis of BMCA is complex and requires a thorough assessment of clinical, pathological, and radiological features. The rarity of BMCA hinders the understanding of its pathogenesis and prognosis [8]. Furthermore, there is currently no established standard treatment regimen [8]. The objective of these two case reports is to enrich the limited literature on this rare and diagnostically challenging tumor, while highlighting its histological and immunohistochemical characteristics along with its differential diagnosis.

## Case presentation

First case 

**Figure 1:**
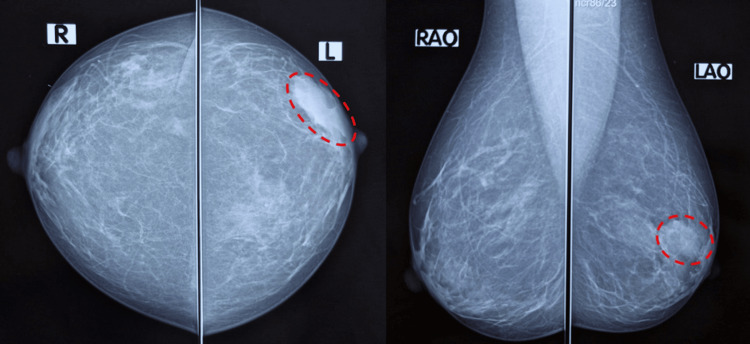
Breast mammography (first case). Radio-opaque opacity of the junction of external quadrants of the left breast (red circle) in cranio-caudal (A) and medio-lateral oblique (B) views.

**Figure 2:**
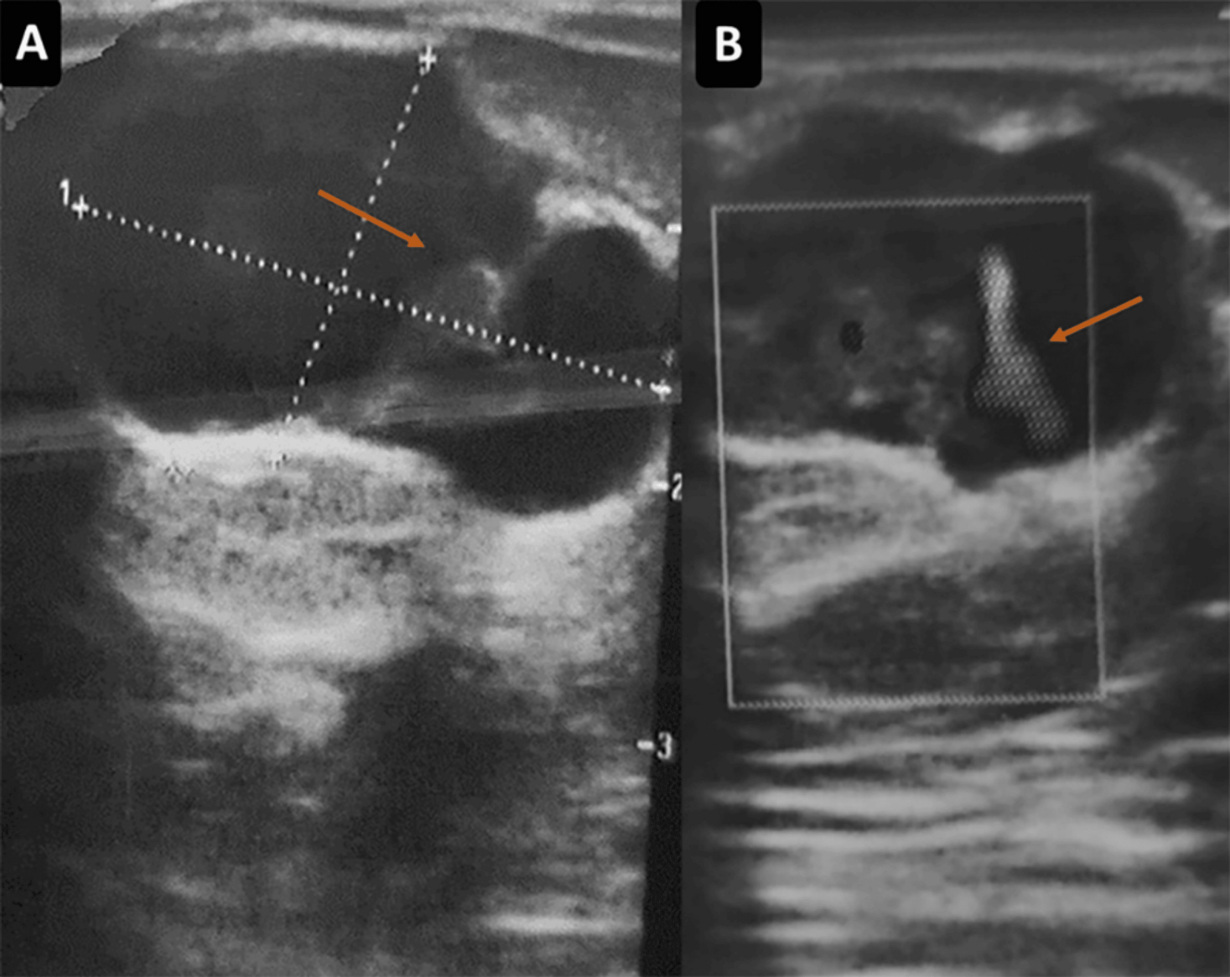
Breast ultrasound (first case). Oval, circumscribed, parallel, predominantly anechoic mass with a tissular component (arrow) (A), demonstrating vascularity on color Doppler (B), measuring 31 × 17 mm.

A 45-year-old patient (G0P0) with no significant medical history presented to the outpatient clinic with a breast lump discovered by self-palpation. Mammography (Figures 1A-1B) and ultrasound (Figures 2A-2B) were performed in April 2023 and revealed two lesions in the left breast: a 31-mm solid-cystic lesion at the junction of the external quadrants and a 33-mm lesion in the upper inner quadrant. There were no suspicious microcalcifications and no axillary adenopathy. The left breast was classified as Breast Imaging-Reporting and Data System (BIRADS) 5 and the right breast as BIRADS 1. 

An ultrasound-guided core micro-biopsy was performed and showed an invasive mammary carcinoma of nonspecific type according to the WHO 2019 classification, grade II (3+2+1) based on the Scarff, Bloom, and Richardson (SBR) score modified by Ellis and Elston, with intra-tumoral lymphocytosis estimated at 3%, without any in situ component, vascular emboli, or perineural invasion. The tumor cells showed a strong ER expression, a weak PR expression, an HER2 negativity, and a Ki-67 proliferation index of 45%. The patient was referred to our institution for further management in May 2023. 

**Figure 3:**
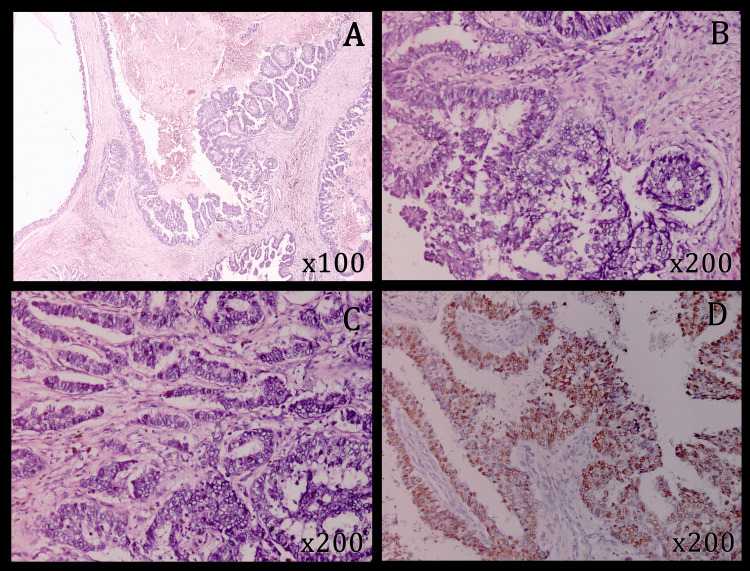
Representative micrographs and immunohistochemical ER profile of the tumor (first case). Tumor exhibits cystic spaces (A) lined by tall columnar cells with moderate cytonuclear atypia, and the presence of intracytoplasmic mucin (B and C). Tumor cells show tufting, stratification, and papillary formation (A, B, and C) and are positive for estrogen receptor (D). (Hematoxylin-eosin: A, ×100; B, C, ×200) (estrogen receptor: D, ×200)

The initial clinical examination found a 4 cm mobile hard mass in the junction of external quadrants of the left breast. After a staging workup showing no secondary lesions, a total left mastectomy was performed. The macroscopic examination found two lesions: the first one, at the junction of the external quadrants, was an indurated, whitish-gray and heterogeneous solid and cystic lesion measuring 30 mm in the major axis. The second one, at the upper inner quadrant, was a 25 mm well-circumscribed, firm and whitish lesion. The axillary lymph node dissection yielded ten lymph nodes, all of which were grossly unremarkable, with no evident enlargement or necrosis. Microscopically, the latter lesion was identified as a fibroadenoma, while the first lesion corresponded to a carcinomatous proliferation showing cystic spaces lined by tall columnar tumor cells with moderate cytonuclear atypia, a mitotic count of 11 mitoses/10 high-power fields (HPFs) (assessed using a microscope with a 0.55 mm field diameter, corresponding to a total area of approximately 2.37 mm²) and the presence of intracytoplasmic mucin (Figures 3A-3C). These tumor cells showed tufting, stratification and papillary formation (Figures 3A-3C). The stroma was fibro-inflammatory, containing numerous deposits of cholesterol crystals. There was no in-situ component, vascular emboli, or perineural invasion. The lateral and deep surgical margins were tumor-free. No lymph node was metastatic. The diagnosis was concluded as invasive mucinous cystadenocarcinoma of the breast (WHO 2019), measuring 3 cm in the major axis, grade II (3+2+2) of SBR modified by Ellis and Elston, classified as pT2N0Mx (American Joint Committee on Cancer (AJCC) 2017, 8th edition). On Immunohistochemistry, the tumor cells were ER-positive (Figure 3D) and negative for PR and HER2.

**Figure 4:**
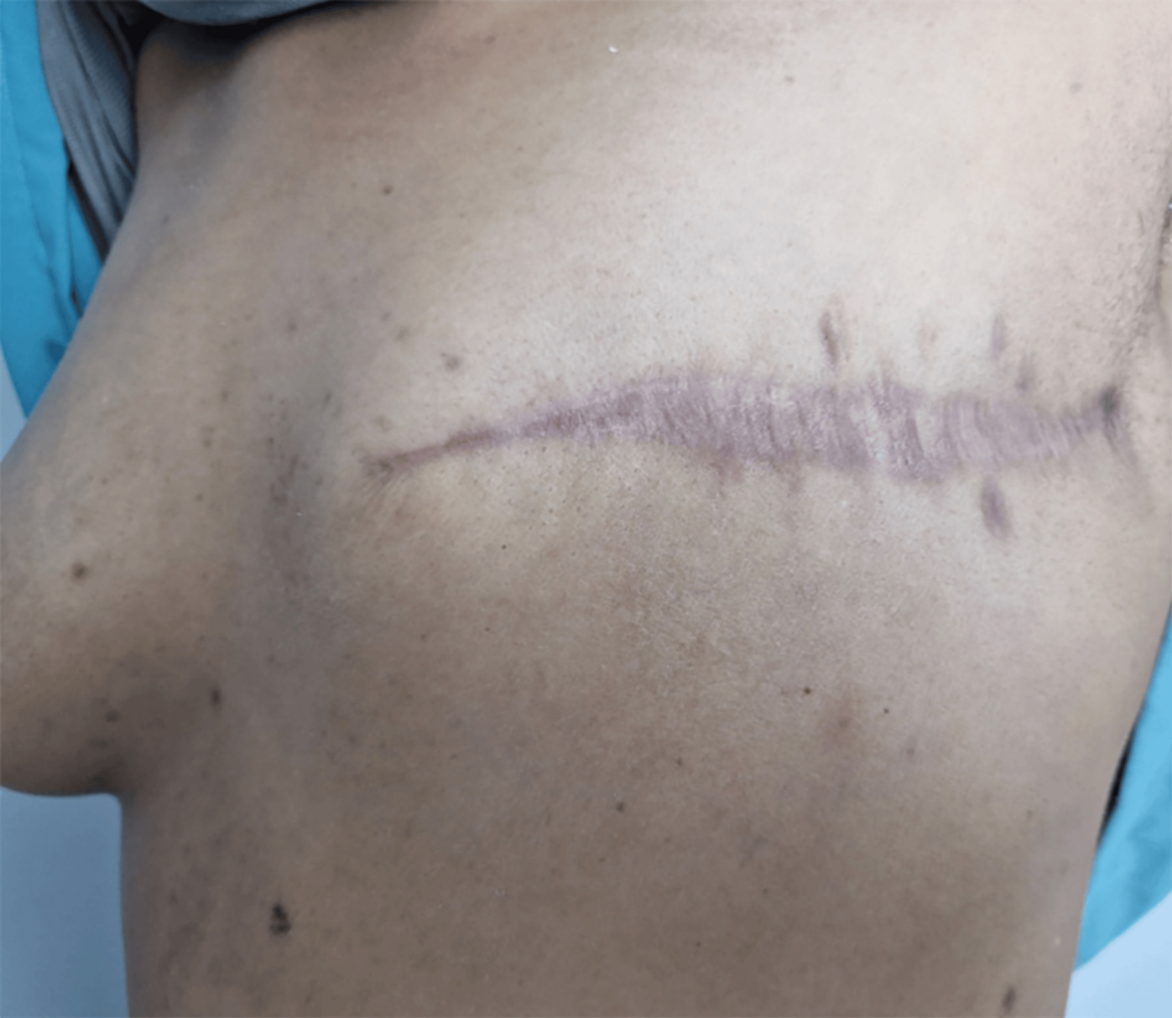
Clinical image after mastectomy (first case). Clean scar with no signs of recurrence on follow-up ultrasound.

The surgical treatment was complemented with adjuvant chemotherapy based on three cycles of Epirubicine plus Cyclophosphamide followed by three cycles of Docetaxel, as decided by a multidisciplinary consultation meeting. The patient evolved favorably (Figure 4). Her next follow-up consultation is scheduled for late December 2025. 

Second case 

A 33-year-old patient (G2P2) with no significant medical history was being followed externally for two nodules located in the inferior and superior outer quadrants of the right breast. These nodules underwent an excision biopsy, and the anatomopathological result showed an invasive mammary carcinoma of nonspecific type according to the WHO 2019 classification, grade III (3+3+3) based on the SBR score modified by Ellis and Elston, with intratumoral lymphocytosis estimated at 15%, without any in situ component, vascular emboli, or perineural invasion. Externally performed mammography and ultrasound showed a suspicious 19 mm tissue nodule in the inferior external quadrant of the right breast associated with a complex cyst measuring 65 mm in the superior external quadrant. There were no suspicious microcalcifications and no axillary adenopathy. The right breast was classified as American College of Radiology (ACR) 4b and the left breast as ACR 2 according to the BIRADS system. The patient was referred to our institution for further management.

**Figure 5:**
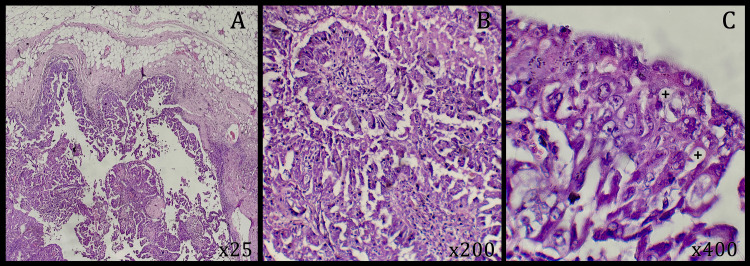
Representative micrographs of the tumor (second case). Tumor proliferation is arranged in papillae and tufts (A-B). Tumor cells are pleomorphic, mucosecretory positive, and show marked cytonuclear atypia (C). (Hematoxylin-eosin: A, ×25; B, ×200; C, ×400)

The initial clinical examination found an 11-cm mass in the external quadrants of the right breast. Magnetic resonance imaging (MRI) was performed, revealing two contiguous masses in the outer quadrants of the right breast. An immunohistochemical examination was conducted on the initial excision biopsy block, which found a triple-negative tumor with negative hormonal receptors and a negative Herceptest. Accordingly, a right total mastectomy was performed. The macroscopic examination found a single mass at the junction of the external quadrants measuring 9 cm in the major axis with a solid-cystic appearance, and upon opening, there was necrotic-hemorrhagic material. The solid part of the mass was friable, brownish in color, and poorly defined. The axillary lymph node dissection found 13 lymph nodes. The microscopic examination showed mammary parenchyma infiltrated by a carcinomatous tumor proliferation arranged in papillae and tufts (Figures 5A-5B). The tumor cells were pleomorphic, muco-secretory, with major cytonuclear atypia and a mitotic count of 45 mitoses/10 HPFs (assessed using a microscope with a 0.55 mm field diameter, corresponding to a total area of approximately 2.37 mm²) (Figure 5C). The stroma was fibro-inflammatory. There was no in situ component, vascular emboli, or perineural invasion. The lateral and deep surgical margins were tumor-free. Two of the 13 examined lymph nodes were metastatic without capsular effraction. The largest metastasis measured 2 cm. The diagnosis was concluded as invasive mucinous cystadenocarcinoma of the breast (WHO, 2019), measuring 9 cm in the major axis, grade III (3+3+3) of SBR modified, classified as pT3N1aMx (AJCC 2017, 8th edition).

To rule out a secondary mammary lesion, a thorough clinical and scannographic examination was performed, which concluded that the lesion was primary mammary. Following a multidisciplinary consultation meeting, the surgical treatment was complemented with radio-chemotherapy based on three cycles of Paclitaxel and one cycle of Epirubicine and Cyclophosphamide. The patient progressed favorably and continues to be followed at our institution.

## Discussion

Significantly less common than its counterparts in the ovary, pancreas, or appendix, BMCA is an exceptionally rare invasive carcinoma officially recognized as a distinct neoplastic entity in the 2019 WHO classification of breast tumors [3]. Therefore, definitive diagnosis is contingent upon excluding metastatic etiology for the lesion [9]. Moreover, BMCA must be distinguished from other mucin-producing mammary tumors [6,9].

BMCA is most commonly observed in perimenopausal and postmenopausal women, aged between 41 and 96 years [2,7,9], with a notable prevalence among Asian women [3]. Patients often present with a palpable breast lump ranging from 0.8 to 19 cm, with an average size of 3 cm [2,10]. It may be associated with symptoms like breast skin ulcers, skin retraction, and nipple discharge [5]. BMCA typically appears as a well-circumscribed, multi-lobular mass with medium-to-high density on mammography, an isoechoic-to-hypoechoic, well-defined mass on ultrasonography, and a well-circumscribed, solid-cystic lesion on MRI [11]. Lesions with cystic and solid components may be radiologically mistaken for papillary neoplasms [11]. 

Macroscopically, BMCA is a well-defined solid and cystic mass. The cystic spaces typically enclose gelatinous material [2]. Gross examination cannot reliably distinguish the tumor from mucinous carcinoma [5].

Histologically, BMCA presents as a well-circumscribed mass featuring cystic spaces lacking peripheral myoepithelial cells and lined by tall columnar cells that exhibit stratification, tufting, and papillary formations towards the lumen [2]. The neoplastic cells have basally located nuclei and contain substantial intracytoplasmic mucin [2]. Mucin is also found within the cystic spaces [2]. The level of cytological atypia varies, even within the same tumor [2], as was shown by the difference in atypia between our two cases. BMCA can occur in a pure form or in combination with other breast cancers, such as ductal carcinoma in situ (DCIS), invasive ductal carcinoma, or invasive pleomorphic lobular carcinoma [6]. A previous study proposed that the coexistence of BMCA with DCIS indicates that mucinous cystadenocarcinoma cells likely originate from mucinous metaplasia of DCIS epithelial cells, associated with the loss of ER and PR expression [12]. Noteworthy, tumors with metaplastic components, including squamous cell carcinoma or high-grade sarcoma, can occur [5]. As illustrated by both cases, evaluating a limited area of the tumor in biopsy samples can lead to misdiagnosis, particularly for uncommon tumors such as BMCA, especially when it is a nonpure form [5]. Immunohistochemically, most BMCA are negative for hormone receptors and HER2 [3]. However, rare cases with ER, PR, and HER2 positivity have been reported [6]. Our first case showed positivity for ER only. Its Ki-67 proliferation index of 45% corroborates previous results, typically high, ranging from 20.5% to 90%, which indicates significant cellular proliferation [5].

Before diagnosing primary BMCA, pathologists must rule out metastases from primary ovarian, pancreatic, or appendiceal mucinous neoplasms by combining clinical, radiological, and pathological findings [7]. Identifying DCIS alongside MCA supports a diagnosis of primary breast carcinoma rather than metastatic disease [7]. BMCA tumor cells are positive for breast tissue lineage markers, including GATA3, GCDFP-15, and mammaglobin. Conversely, they are negative for WT1, villin, TTF1, PAX8, CDX2, and SATB2, helping to rule out other potential primary sites [7]. Additionally, the CK7/CK20 combination can be helpful. Although focal CK20 positivity was reported in rare cases, most BMCAs are CK7-positive and CK20-negative [7]. This contrasts with ovarian and pancreatic mucinous carcinomas, which often show co-expression of CK7 and CK20, while gastrointestinal mucinous neoplasms typically exhibit CK20 positivity alone [6-8].

When considering the differential diagnosis of BMCA, several primary breast lesions must be excluded, including mucocele-like lesions, mucinous carcinoma, encapsulated papillary carcinoma, and invasive papillary carcinoma (IPC). The triple-negative phenotype and intracellular mucus help rule out the last three diagnoses, knowing that IPC has no to minimal mucin production [6-8]. Unlike mucocele-like lesions, BMCA is characterized by the presence of mucinous and heterologous cells, its invasive behavior, and the loss of myoepithelium [6-8].

Despite its triple-negative immune profile, BMCA generally has a favorable prognosis. No disease-related deaths have been reported to date [5]. There have been two documented cases of local recurrence and one distant metastasis [5,6]. Axillary lymph node involvement is rare, with no cases reported involving more than three metastatic lymph nodes [5,7]. Nevertheless, the follow-up period was relatively short, ranging from 3 to 108 months (median: 12 months), and is insufficient to fully determine the tumor's biological behavior [6].

Molecularly, gene analyses of two cases identified recurrent mutations in PIK3CA, KRAS, MAP2K4, RB1, KDR, PKHD1, TERT, and TP53. Nonetheless, knowledge about the genetic alterations in BMCA is still limited [8], hindering optimal management strategies.

Standardized or targeted treatments for BMCA remain unavailable. Partial or radical mastectomy has been performed in all reported cases, with chemotherapy and radiotherapy being used selectively [7], while hormone therapy and HER2-targeted therapy were applied for rare hormone receptor-positive and HER2-positive tumors [7].

The present report provides valuable clinical and pathological insights into BMCA; however, it is not without limitations. The small number of cases and the relatively short follow-up restrict the generalizability of the clinical, pathological, and therapeutic observations. In addition, detailed clinical and macroscopic data were limited, as the initial assessments and biopsies were performed externally, and no macroscopic images were available. Furthermore, molecular and genetic information on BMCA remains scarce, and standardized treatment guidelines have yet to be established due to the tumor’s extreme rarity. These limitations underscore the need for larger studies with extended follow-up to better characterize the behavior and optimal management of this rare neoplasm.

## Conclusions

BMCA is an uncommon primary breast cancer. Its differential diagnosis includes other mucin-producing breast tumors and metastatic mucinous cystadenocarcinoma, particularly from pancreatic, appendiceal, or ovarian origins. Despite its triple-negative profile and high proliferative index, BMCA generally has a favorable prognosis, with no reported disease-related deaths. However, its etiopathogenesis remains unclear, and further studies are needed to establish standardized diagnostic and treatment guidelines.
